# Heparin binding proteins on monocyte cell surfaces regulates pre-inflammatory responses in diabetes

**DOI:** 10.46439/allergy.4.037

**Published:** 2023

**Authors:** Andrew Jun Wang, Aimin Wang, Vincent Hascall

**Affiliations:** 1 Department of Biomedical Engineering, Cleveland Clinic, Cleveland, Ohio 44195, USA

**Keywords:** Diabetes, Heparin, Inflammation, Hyaluronan, Hyperglycemia, U937 pre-monocytes, Heparin binding protein

## Abstract

Many diabetic complications, such as renal and cardiovascular disease, share a common association with extensive and chronic inflammation due to infiltration by activated leukocytes that originate from the bone marrow (BM). Our previous study demonstrated that macrophage progenitor cells that divided in hyperglycemia induced intracellular synthesis of hyaluronan and became pro-inflammatory macrophages (Mpi), and that the presence of low concentrations of heparin (~50 nM) prevented the intracellular HA synthesis and promoted the formation of tissue repair macrophages (Mtr). However, the molecular mechanism underlying heparin’s role is still unknown. This study showed that heparin can be internalized by dividing monocyte progenitor cells. Further, there are two most abundant heparin binding proteins, alpha-enolase (ENO-1) and cofilin-1, identified on monocyte cell surfaces. In addition to their conventional roles inside of cells, ENO-1 and cofilin-1 can be found on cell surfaces and are also involved in autoimmune diseases. Thus, this study provides new insight into heparin’s role in regulating monocyte and macrophage function.

## Introduction

Diabetes, a disease that features hyperglycemia, is a major health epidemic at this time. It affects ~30 million people in the USA (9.4% of the population) according to the 2017 National Diabetes Statistics Report from the Centers for Disease Control and Prevention (CDC). Nearly 34% of the adult population is hyperglycemic enough to be considered pre-diabetic [1]. Many diabetic complications, such as renal and cardiovascular disease, share a common association with extensive and chronic inflammation due to infiltration by activated leukocytes that originate from the bone marrow (BM). A recent comprehensive review article describes alterations to blood cells associated with diabetes mellitus (DM) pathologies [2]. It concludes: “This review expands our current understanding of the role of DM in risk of development of blood cell damages. However, precise mechanism of DM action that stimulates these damages remains unclear.”

Hyperglycemia in diabetes induces impairment of hematopoiesis, an important consequence in BM that contributes to chronic complications in advanced diabetes. Euglycemic control in diabetes minimally reverses the hematopoietic stem cell (HSC) and hematopoietic progenitor cell (HPC) defects [3]. A mechanism of central importance is an increased ratio of Mpi/Mtr monocytes/macrophages in diabetic BM in both animal models and humans [4–11]. This appears to have key roles in inducing inflammatory processes that are poorly understood [6,9,10,12–17]. Further, non-steroidal anti-inflammatory drugs (NSAIDs) do not reverse diabetic BM dysfunctions. Understanding the formation of the pro-inflammatory Mpi monocytes and macrophages in diabetic BM and the reason for their NSAID resistance is an important goal for future studies [12]. Our recent publication indicates that aberrant intracellular synthesis of hyaluronan (HA) by hyperglycemic dividing BM progenitors is the central mechanism involved [18]. This study demonstrated that macrophages that divided from bone marrow progenitor cells in hyperglycemia are pro-inflammatory (Mpi) and that the presence of low concentrations of heparin (~20 nM) prevented the intracellular HA synthesis and promoted a tissue repair (Mtr) phenotype. This provides evidence that heparin can potentially target hyperglycemic hematopoietic cell alteration by preventing HSC and/or HPC differentiation to pro-inflammatory Mpi monocytes/macrophages with loss of function, and by promoting tissue repair anti-inflammatory Mtr monocytes/ macrophages. However, the cell surface receptor(s) of heparin on monocytes/macrophages that regulate hyperglycemic glucose induced responses in dividing cells is still unclear.

Our previous study showed that heparin was uptaken by mesangial cells into intracellular compartments and then into the nucleus [19]. We have also identified two cell surface proteins on mesangial cells that bound to a novel biotin labeled heparin that did not alter the role of heparin in regulating the hyperglycemia induced responses [19]. Using a similar strategy to the one described for the mesangial cells [19], this study determined to what extent heparin is taken up by U937 pre-monocytes and what molecular mechanism underlies the interaction between heparin and monocyte cells that mediates heparin’s role in inhibiting the inflammatory responses of circulating monocytes formed during hyperglycemia. We have identified that alpha-enolase and cofilin-1 are two receptor proteins on monocyte cell surface for heparin binding to the cells.

## Methods

### Culturing U937 pre-monocyte cells

U937 cells (ATCC CRL-1593.2, a human leukemic cell line) were cultured in RPMI 1640 medium (1 g/liter glucose, 5% fetal bovine serum (FBS)), serum starved (0.5% fetal bovine serum) for 24 hrs [18] and then treated in the different media before the cells were harvested and separated from their media for various assays.

### Immunohistochemistry

To determine whether or not heparin was internalized by U937 pre- monocytes, growth-arrested cultures were stimulated to divide with 10% FBS in high glucose medium in the presence of 1 μg/ml fluorescein isothiocyanate (FITC)-heparin for 1 hr, and for 24 hrs. At the end of each incubation period, U937 cells from different treatments were transferred to glass slides via cytopsins. The cytospins were air-dried and then lightly fixed by 4% paraformaldehyde for 10 min at room temperature [18]. Vectashield mounting medium with 4’,6- diamidino-2-phenylindole (DAPI) (H-1200; Vector Laboratories) was applied, followed by coverslip application. Cell samples were then analyzed by fluorescent microscopy. The images shown represent the results from two different experiments.

### Isolation of heparin binding proteins from U937 pre-monocyte cell surfaces

Quiescent cells grown on 150 cm^2^ plates were washed three times with ice-cold phosphate-buffered saline (PBS) and cooled to 4°C for 30 min in PBS. 20 ml of precooled heparin-biotin at 1 μg/ml in PBS containing 0.1 % FBS and 5 mM MgCl_2_ [19,20] was added to each plate in the presence or absence of a 100-fold excess of unlabeled heparin. After incubation on ice, medium was collected. The bound cells were washed five times with 50 ml of PBS and then solubilized in 6 ml of radioimmunoprecipitation assay (RIPA) buffer containing proteinase inhibitors. Nonspecific binding was determined with inclusion of a 100-fold excess of unlabeled heparin [19]. Heparin- Biotin-protein complexes in this extract were isolated with the Pierce Monomeric Avidin Kit according to the manufacturer’s instruction. Briefly, 2 ml of RIPA extracts were applied to a prepacked monomeric avidin column (2 ml) equilibrated in PBS. After washing with 12 ml of PBS, bound material was eluted with 12 ml of biotin blocking and elution buffer. This eluate was concentrated by Amicon Ultra-0.5 Centrifugal Filter Unit against an Ultracel-3 regenerated cellulose membrane [19].

The concentrated samples were analyzed by electrophoresis under denaturing conditions on precast polyacrylamide 4 to 20% gradient gels according to the method of Laemmli [21]. After electrophoresis, gels were stained with GelCode Blue Stain Reagent (Thermo Fisher Scientific) and air dried.

### Protein analysis with LC-MS

For the protein digestion, the bands were cut to minimize excess polyacrylamide and divided into a number of smaller pieces. The gel pieces were washed with water and dehydrated in acetonitrile. The bands were then reduced with DTT and alkylated with iodoacetamide. All bands were then digested in-gel using trypsin, by adding 5 μl of trypsin (10 ng/μl) in 50 mM ammonium bicarbonate and incubating overnight at room temperature to achieve complete digestion. The peptides that were formed were extracted from the polyacrylamide in two aliquots of 30 μl of 50% acetonitrile with 5% formic acid. These extracts were combined and evaporated to <10 μl in Speedvac and then resuspended in 1% acetic acid to make a final volume of ~30 μl for LC-MS analysis [19].

The LC-MS system was a Finnigan LTQ-Obitrap Elite hybrid mass spectrometer system. The HPLC column was a Dionex 15 cm x 75 μm id Acclaim Pepmap C18, 2 μm, 100 Ǻ reversed-phase capillary chromatography column. Five μL volumes of the extracts were injected, and the peptides were eluted from the column by an acetonitrile/0.1% formic acid gradient at a flow rate of 0.25 μl/min. They were then introduced into the source of the mass spectrometer online. The microelectrospray ion source is operated at 2.5 kV. The digests were analyzed using the data dependent multitask capability of the instrument acquiring full scan mass spectra to determine peptide molecular weights and product ion spectra to determine amino acid sequence in successive instrument scans. The data were analyzed by using all collision-induced dissociation spectra collected in the experiment to search the human SwissProtKB database with the Sequest search programs [19].

## Results

### Internalization of heparin by monocytes

**Figure 1.**
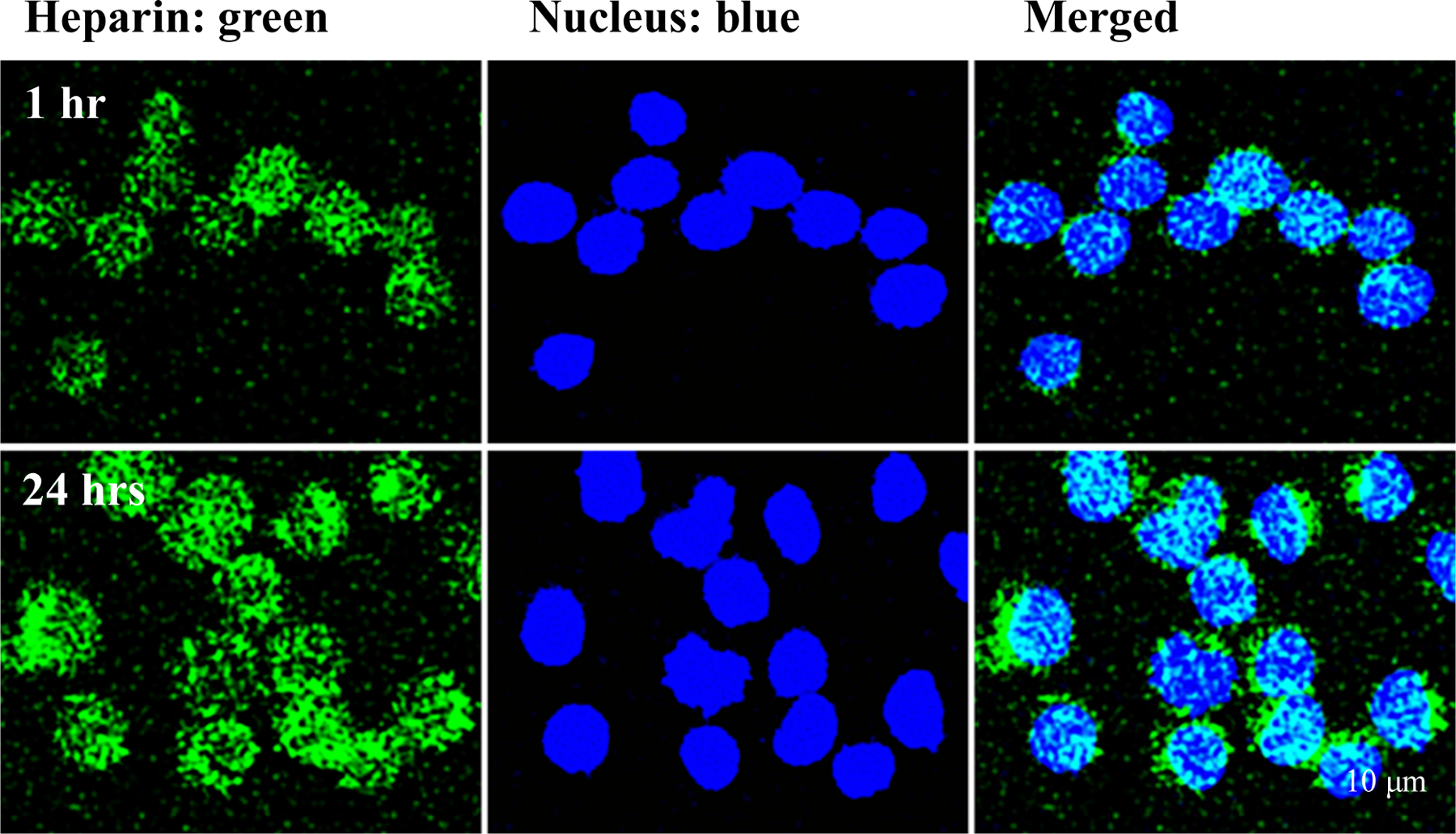
Fluorescent cell imaging of cultured U937 pre-monocytes for heparin internalization. The cells were initiated to divide from G0/G1 in hyperglycemic medium in the presence of 1 μg/ml of fluorotagged FITC heparin (green), and nuclei were stained by DAPI (blue). The cell imaging was done by fluorescent confocal microscopy at 1 hour and 24 hours. Internalization of the fluorotagged heparin into the cytoplasm is apparent.

Our previous studies [18] have shown that heparin at 1 μg/ml can prevent the formation of pro-inflammatory monocyte/ macrophages from the progenitor cells (such as U937 cells) induced by hyperglycemia and promote a tissue repair (Mtr) phenotype. This provides evidence that a direct interaction occurred between heparin and the monocyte progenitor cells. We then determined whether this direct interaction of heparin could lead to its internalization by monocyte progenitor cells. Serum starved U937 cells, a monocytic cell line, were stimulated to divide by 5% FBS in the presence of 1 μg/ml of fluorotagged heparin [19,22,23]. Figure 1 shows that U937 cells internalize fluorotagged heparin within 1 hour, which is maintained for 24 hours through cell division. This indicates that the heparin was internalized shortly after the cells initiated division, similar to our finding in cultured rat mesangial cells [19]. Also, these results suggest that heparin not only could act on cell surfaces via a receptor mediated pathway to regulate the responses of dividing cells induced by hyperglycemia, but also could directly act intracellularly to regulate some further gene expressions and cellular responses of the dividing cells. Further, these results and their tight binding provide strong evidence for a cell surface receptor on dividing U937cells [24].

### Isolation of cell surface heparin binding proteins

Our previous studies [22, 23] have shown that heparin induces a robust formation of an extracellular HA matrix with a Kd of ~20 nM while blocking 1) the intracellular HA responses and 2) the formation of pro-inflammatory monocytes in hyperglycemic dividing the progenitor cells, and promoting a tissue repair (Mtr) phenotype. Further, a specific binding of heparin to monocytes (U937 cells) has been carefully documented in the previous study [25]. These results provide compelling evidence for a cell surface receptor(s) on dividing progenitor cells including U937 cells. Therefore, biotinylated heparin was used to test this possibility. In order to identify the cell surface heparin binding proteins, serum starved U937 cells were incubated with 1 μg/ml of biotinylated heparin in the presence or absence of 100 μg/ml heparin at 4°C for one hour [19]. Then the cell layers were washed three times with cold binding buffer, and the RIPA buffer was used to extract protein samples.

**Figure 2.**
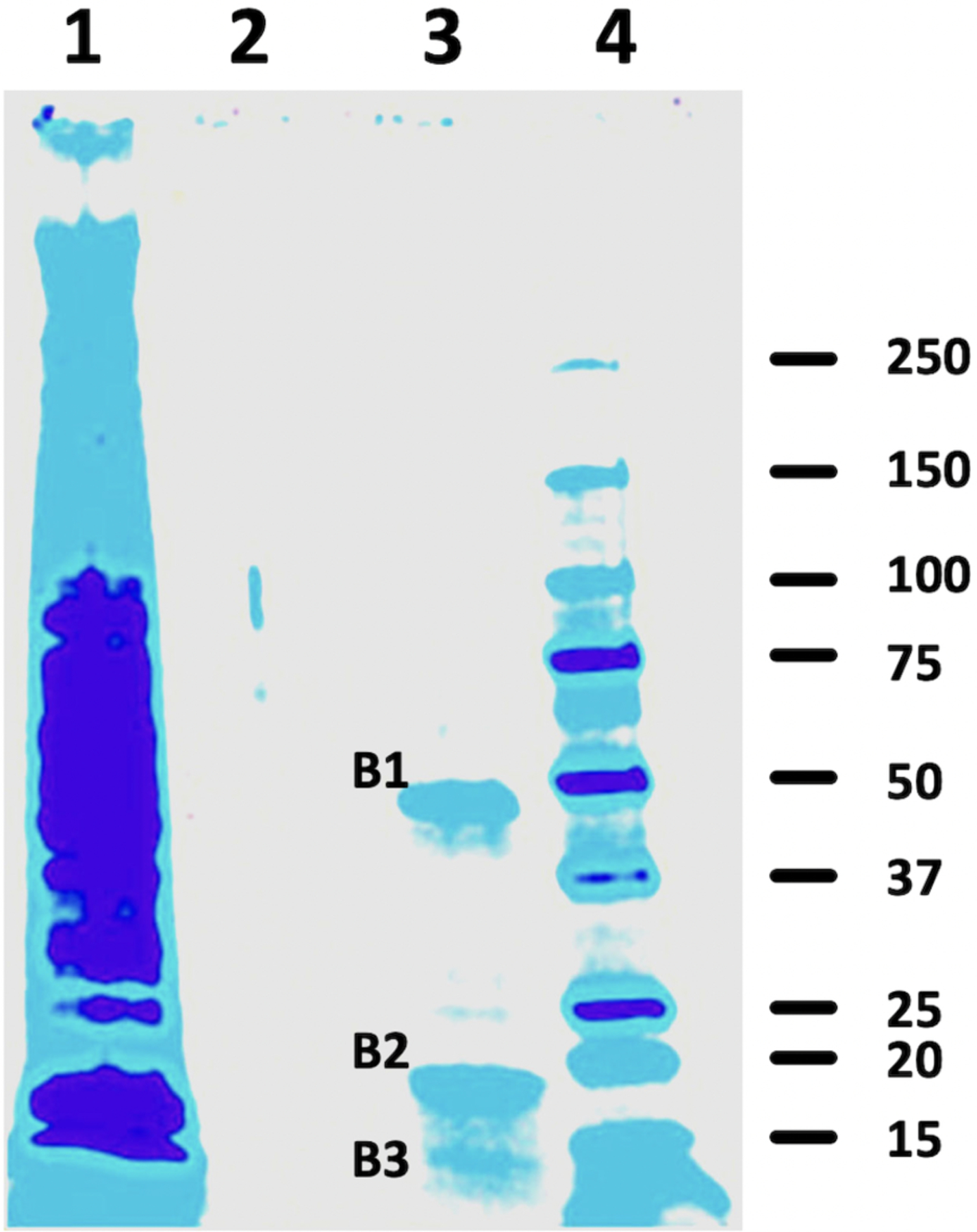
Heparin binding proteins on cell surfaces of G0/G1 U937 pre-monocytes. Bands that bind biotinylated heparin (lane 3) were absent when excess unlabeled heparin was used as a competitor (lane 2). Sample in lane 1 is the total initial RIPA protein from U937 cells. Mass- Spec analysis of trypsin digests of the 3 bands in lane 3 identified alpha-enolase and cofilin-1 as the two most abundant heparin binding proteins.

Figure 2 shows an SDS-PAGE gel with an aliquot of the total cellular fraction (lane 1), the excess heparin specificity control (lane 2), and the biotinylated heparin alone (lane 3). Two major (B1 and B2) and one minor (B3) bands were found in lane 3 that were absent in lane 2, providing evidence that the biotinylated heparin specifically bound to these proteins on the cell surface and selectively extracted them from the plasma membrane (lane 1). The apparent molecular weights for two major bands, B1 and B2, were ~47.4 and ~17.5 kDa respectively, and one for B3 was ~13.6 kDa.

### Mass Spec analysis of biotinylated heparin binding proteins from U937 cells

The three bands were excised from the gel (Figure 2, lane 3). These bands were washed, reduced, alkylated, and digested with trypsin. The digests were analyzed by a capillary column LC- tandem MS, and the CID spectra were searched with the human SwissProtKB database. All of these analyses utilized an LC gradient from 2 to 70% acetonitrile in 45 minutes. The results are summarized in Table 1. The most abundant non-keratin protein in band B1 was alpha-enolase (~47.1 kDa), which was identified by 25 peptides covering 51% of the protein sequence with a Sequest Score of 355.12. The other likely minor non-keratin proteins were elongation factor 1-alpha 1 (~50.1 kDa) identified by 15 peptides covering 34% of the protein sequence with a Sequest Score of 161.2, and serpin H1 (~46.4 kDa) by 10 peptides covering 23% of the protein sequence with a Sequest Score of 80.31. In band B2, the most abundant non-keratin protein was identified as cofilin-1 (~18.5 kDa) by the presence of 20 peptides covering 83% of the protein sequence with a Sequest Score of 357.53. The other minor non-keratin candidates were cofilin-2 (~18.7 kDa) identified by 9 peptides covering 55% of the protein sequence with a Sequest Score of 141.68 and destrin (~18.5 kDa) by 12 peptides covering 44% of the protein sequence with a Sequest Score of 88.8.

In the minor band B3, the most abundant non-keratin protein identified was albumin (~69.3 kDa). The albumin was also identified as minor components in bands 1 and 2. However, this is not surprising result because bovine serum albumin has been used in heparin binding buffer to reduce the non-specific binding of heparin to cell surface proteins [26]. This result is consistent with the previous study that showed that heparin can form the complex with albumin [26]. Importantly although albumin can bind to heparin, the binding site of albumin on the heparin must be different from ones that bind to the proteins we have identified here.

In summary, three bands were cut from a dry Coomassie blue stained 1D gel, and the two most abundant heparin binding proteins identified were alpha-enolase and Cofilin-1. There were several less abundant proteins identified with a sequence covering of more than 20% and a sequest score of more than 80. These likely candidates were the minor components including elongation factor 1-alpha 1, serpin H1, cofilin-2, and destrin.

## Discussion

This study was undertaken to determine the heparin binding proteins on the U937 cells using the biotinylated heparin that has been successfully used to determine the cell surface heparin binding proteins on mesangial cells [19]. On the U937 cell surface, the two most abundant heparin binding proteins identified on the growth arrested U937 cells were alpha-enolase and cofilin-1 along with other heparin binding proteins such as elongation factor 1-alpha 1 and serpin H1. Interestingly, these are surprising results because 1) the conventional locations of these proteins are in the intracellular compartments, 2) each of them has important roles in glucose metabolism (alpha- enolase), protein synthesis (elongation factor 1-alpha 1), actin polymerization (cofilin-1,2 and destrin) and a molecular chaperone in ER (serpin H1), and 3), their heparin-binding properties have not been broadly recognized. However, the findings are indeed consistent with the previous studies in the literature that they can bind to heparin [27–29].

The well-known function of alpha-enolase is its role in mediating glycolysis that occurs in the cytosol and catalyzes the conversion between 2-phospho-glycerate and phosphoenolpyruvate [30]. The novel roles of this protein have been well documented in 1) cell cycle, 2) myogenesis, 3) cardiomyocyte contraction, and 4) formation of chaperon pathways [30,31]. It facilitates glycolysis of glucose to affect proliferation, angiogenesis, and metastasis formation as described in Warburg effects [31,32] in previous studies. Interestingly, previous studies have shown that inflammatory stimuli such as lipopolysaccharide (LPS) rapidly up-regulated cell-surface expression of alpha-enolase on human blood monocytes and U937 monocytic cells by rapid translocation of alpha-enolase to the cell surface from the cytosol [31,33], which promotes monocytes into the inflamed lung [34].

On the cell surface, alpha-enolase has been shown to act as a receptor for plasminogen binding in the formation of serine protease plasmin from plasminogen (PLG) under physiological stimuli such as urokinase-type PLG activator (uPA) or tissue-type PLG activator (tPA) [35]. This promotes extracellular matrix degradation and subsequent pathogens, immune cell and cancer cell invasion into tissue that leads to infection, inflammation and metastasis formation [31, 32, 35]. Thus, alpha-enolase can act as a receptor on the cell surface mediating bacteria adherence and internalization that leads to infection and the efficient dissemination of bacteria [31]. Such a mechanism has been proposed in the lung epithelial cell infection by bacteria, in which bacteria and epithelial cell surface enolases interact with each other via the RNA that serves as a bridge molecule thereby facilitating the pathogen dissemination [31]. Moreover, previous studies have shown that the enolase can be secreted extracellularly either in the form of an exosome or as a free molecule [31], and in this case, it can induce the migration of immune cells and promote the proinflammatory phenotype of immune cells [31,35].

Another most abundant protein identified was cofilin-1. Again, the main function of cofilin-1 is in the intracellular compartment as an actin modulating protein to depolymerize the F-actin and to inhibit G-actin polymerization [36]. Further, cell surface actin has been shown to be involved in the plasminogen binding and activation, suggesting that the actin binding protein such as cofilin is directly involved in such interactions [37]. Thus, future studies need to be done to determine whether the cofilin is also required for the plasminogen binding and activation on the cell surface. In addition, along with alpha-enolase as well as other identified heparin binding proteins in this study such as elongation factor 1-alpha 1 and serpin H1, cofilin-1 has been shown to be involved in an autoantibody-induced disease [38–43].

Previous studies have shown that these autoantibodies have been found in diseases such as rheumatoid arthritis (RA), Systemic lupus erythematosus (SLE), Sjögren’s syndrome (SjS), mixed connective tissue disease (MCTD), systemic sclerosis (SSc), Alzheimert’s disease, cancer and/or non-specific idiopathic pneumonia [38–43]. In rheumatoid arthritis, ENO-1 can be reattached to the cell surface of monocytes, which induces the CD14-dependent TLR4 signaling to stimulate their pro-inflammatory state [31]. Such a reattachment of ENO-1 to monocyte surface has been found in patients with a kidney stone disease [31].

Particularly pertinent to this study, an interesting previous study showed that heparin binds to the cell surface GRP78 to interrupt the interaction between GRP78 and its autoantibody, which attenuates its role in stimulating tumor growth in prostate cancer [44]. Thus, we propose that heparin binds to the cell surface of monocytes that initiates signaling to regulate the inflammatory responses [18].

Lastly, previous studies have shown that there is a heparin binding site on monocyte cell surfaces [24] and that alpha-enolase is the major plasminogen receptor on the U937 cell surface [33–45], consistent with the findings observed in this study. We propose: 1) that alpha-enolase was expressed and then translocated onto the cell surface, 2) that heparin binds to cell surface alpha-enolase and then is endocytosed into the cytosol and into the nucleus to regulate gene expression and inflammatory responses, and 3) that by removing alpha-enolase from the cell surface heparin directly blocks the plasminogen activation, which mitigates the pro-inflammatory monocyte infiltration into inflamed tissues. Altogether, these data open a new avenue to study the molecular mechanism(s) underlying the role of heparin in inhibiting pro-inflammatory responses in monocytes and macrophages in diabetes and autoimmune diseases.

## Figures and Tables

**Figure 3.**
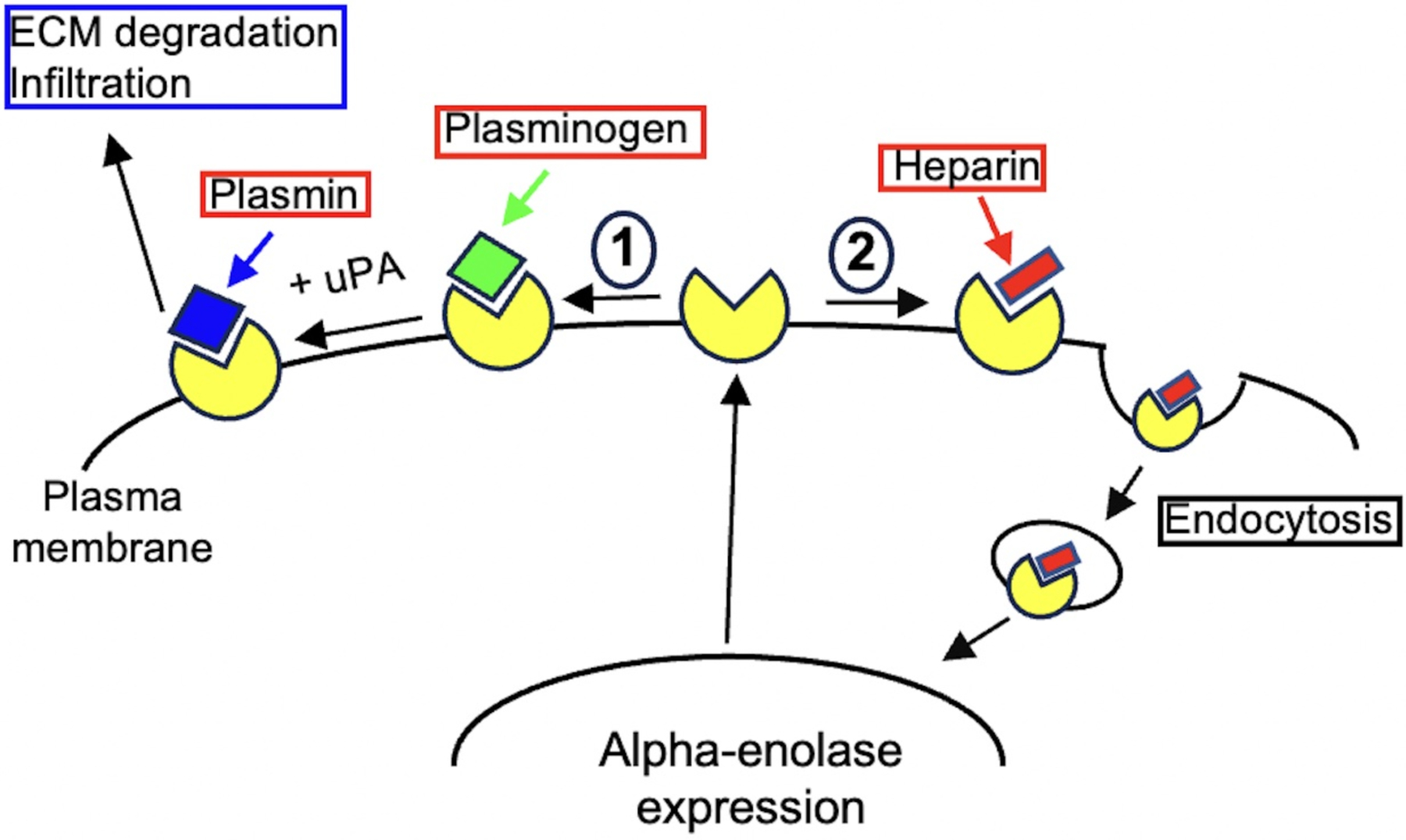
The proposed model for the roles of alpha-enolase and its interaction with heparin on cell surface. 1) Plasminogen binds to alpha-enolase on the cell surface where it is activated by uPA to form plasmin that degrades the extracellular matrix to facilitate proinflammatory monocyte infiltration into the inflamed tissues. 2) Heparin binds to alpha-enolase on the cell surface that mediates heparin’s internalization into cytosol to regulate cellular responses and then into nucleus to regulate the gene expression.

**Table 1.** Heparin binding proteins*.

Sample name	Sample number	Protein	Accession number	Mass kDa	Peptides #	Sequence Cov (%)	Sequest Score
Band 1	SB15–34-16	Alpha-enolase	P06733	47.1	25	51	355.12
		Elongation factor 1-alpha 1	P68104	50.1	15	34	161.2
		Serpin H1	P50454	46.4	10	23	80.31
Band 2	SB15–34-17	Cofilin-1	P23528	18.5	20	83	357.53
		Cofilin-2	Q9Y281	18.7	9	55	141.68
		Destrin	P60981	18.5	12	44	88.8
Band 3	SB15–34-18	Albumin	P02768	69.3	15	26	121.99

**Note:** Proteins with Sequence coverages of more than 20% and Sequest Scores of more than 80 are listed.

## Data Availability

The datasets used and/or analyzed during the current study are available from the corresponding author on reasonable request.
